# Piezoelectric Microvibration Mitigates Estrogen Loss-Induced Osteoporosis and Promotes Piezo1, MicroRNA-29a, and Wnt3a Signaling in Osteoblasts

**DOI:** 10.3390/ijms22179476

**Published:** 2021-08-31

**Authors:** Re-Wen Wu, Wei-Shiung Lian, Yu-Shan Chen, Jih-Yang Ko, Shao-Yu Wang, Holger Jahr, Feng-Sheng Wang

**Affiliations:** 1 Department of Orthopedic Surgery and Chang Gung University College of Medicine, Kaohsiung Chang Gung Memorial Hospital, Kaohsiung 83301, Taiwan; ray4595@cgmh.org.tw (R.-W.W.); kojy@cgmh.org.tw (J.-Y.K.); 2 Core Laboratory for Phenomics and Diagnostic, Department of Medical Research, Chang Gung University College of Medicine, Kaohsiung Chang Gung Memorial Hospital, Kaohsiung 83301, Taiwan; lianws@gmail.com (W.-S.L.); ggyy58720240@gmail.com (Y.-S.C.); vip690221@gmail.com (S.-Y.W.); 3 Center for Mitochondrial Research and Medicine, Kaohsiung Chang Gung Memorial Hospital, Kaohsiung 83301, Taiwan; 4 Department of Anatomy and Cell Biology, University Hospital RWTH Aachen, 52074 Aachen, Germany; h.jahr@masstrichtuniversity.nl; 5 Department of Orthopedic Surgery, Maastricht University Medical Center, 6229 ER Maastricht, The Netherlands

**Keywords:** piezoelectric microvibration, osteoporosis, Piezo1, Wnt3a, Dkk1, microRNA-29a

## Abstract

Biophysical stimulation alters bone-forming cell activity, bone formation and remodeling. The effect of piezoelectric microvibration stimulation (PMVS) intervention on osteoporosis development remains uncertain. We investigated whether 60 Hz, 120 Hz, and 180 Hz PMVS (0.05 g, 20 min/stimulation, 3 stimulations/week for 4 consecutive weeks) intervention affected bone integrity in ovariectomized (OVX) mice or osteoblastic activity. PMVS (120 Hz)-treated OVX mice developed fewer osteoporosis conditions, including bone mineral density loss and trabecular microstructure deterioration together with decreased serum resorption marker CTX-1 levels, as compared to control OVX animals. The biomechanical strength of skeletal tissue was improved upon 120 Hz PMVS intervention. This intervention compromised OVX-induced sparse trabecular bone morphology, osteoblast loss, osteoclast overburden, and osteoclast-promoting cytokine RANKL immunostaining and reversed osteoclast inhibitor OPG immunoreactivity. Osteoblasts in OVX mice upon PMVS intervention showed strong Wnt3a immunoreaction and weak Wnt inhibitor Dkk1 immunostaining. In vitro, PMVS reversed OVX-induced loss in von Kossa-stained mineralized nodule formation, Runx2, and osteocalcin expression in primary bone-marrow stromal cells. PMVS also promoted mechanoreceptor Piezo1 expression together with increased microRNA-29a and Wnt3a expression, whereas Dkk1 rather than SOST expression was repressed in MC3T3-E1 osteoblasts. Taken together, PMVS intervention promoted Piezo1, miR-29a, and Wnt signaling to upregulate osteogenic activity and repressed osteoclastic bone resorption, delaying estrogen deficiency-induced loss in bone mass and microstructure. This study highlights a new biophysical remedy for osteoporosis.

## 1. Introduction

Osteoporosis is one major risk factors of musculoskeletal frailty in senile patients [1]. Bone formation and remodeling dysregulation increases bone turnover, which aggravates bone mass loss and microarchitecture damage, accelerating the development of osteoporosis [2,3]. Bone-forming cells construct sophisticated mineralized microstructure and couple with osteoclasts to harmonize skeletal tissue homeostasis and microenvironment integrity [4]. Expanding evidence has revealed that the Wnt signaling pathway and Wnt inhibitors, including Dickkopf-1 (Dkk1) and sclerostin (SOST), are important to regulate osteoblastic activity and bone formation [5]. On the other hand, receptor activator of nuclear factor kappa-B ligand (RANKL) steer monocytes or macrophages into osteoclast lineages and promotes bone resorption activity, whereas osteoprotegrin (OPG) represses osteoclast formation during osteoporosis [6].

Maintenance of bone tissue homeostasis through controlling osteoblast metabolism or osteoclast function is advantageous to slow down bone loss [7]. Accumulating studies have established a plethora of pharmaceutical and biochemical strategies, including bone anabolic agents [8] and osteoclast inhibitors [9], to decrease the risk of human osteoporosis or osteoporotic fracture. Metabolites [10], short-chain fatty acids [11] or probiotics [12] supplements prevent age-induced osteoporotic skeleton development in mice. In addition, regular workouts promote myokine secretion, which correlates with a low risk of human osteoporosis [13] and attenuates osteoporosis in ovariectomized mice [14] and in limb disused animals [15], hinting that physical activity or biophysical stimulation may promote skeletal tissue anabolism and microstructure integrity.

Bone-forming cells convert extracellular dynamic physical or mechanical stimuli into intracellular signaling through mechanical sensing molecules, like Piezo1, integrin or gap junction proteins [16]. Of stimuli, mechanical loading downregulates angiogenic activity and bone remodeling in ovariectomized mice [17]. Low intensity mechanical vibration produces oscillatory force which influences cell activity and tissue function. Whole-body vibration improves bone mineral density of tibia in postmenopausal women [18]; however, a study by Liphardt et al. reveals that the bone quality of postmenopausal women with osteopenia is unimproved upon whole-body vibration for 12 months [19]. In experimental animals, low magnitude whole-body vibration represses osteoblast senescence and downregulates bone mass loss in aged mice [20] and promotes articular cartilage thickness in high fat diet-induced obese mice [21]. Piezoelectric vibration generates vibratory stimuli through piezoelectric transducers. Low pulse vibration (0.25 N, 30 Hz) for 48 h is found to increase IL-4, IL-12, TGF-β1, and OPG production in murine osteoblasts. [22]. The effects of piezoelectric microvibration stimulation (PMVS) on estrogen deficiency-induced bone loss or osteoblastic activity using very low vibration amplitude remains uncharacterized. We hypothesized that PMVS intervention may attenuate osteoporosis and activate Piezo1 or Wnt signaling to promote osteoblast function.

This study aimed to utilize a PMVS device and investigate whether bone mass, biomechanics, or osteogenic differentiation was affected in ovariectomized mice upon PMVS intervention and examined whether osteoblastic activity, mechanoreceptor Piezo1, Wnt3a, or microRNA-29a signaling was altered in PMVS-treated MC3T3-E1 osteoblasts.

## 2. Results

### 2.1. PMVS Intervention Downregulated Serum Bone Resorption Marker CTX-1 Levels

**Figure 1:**
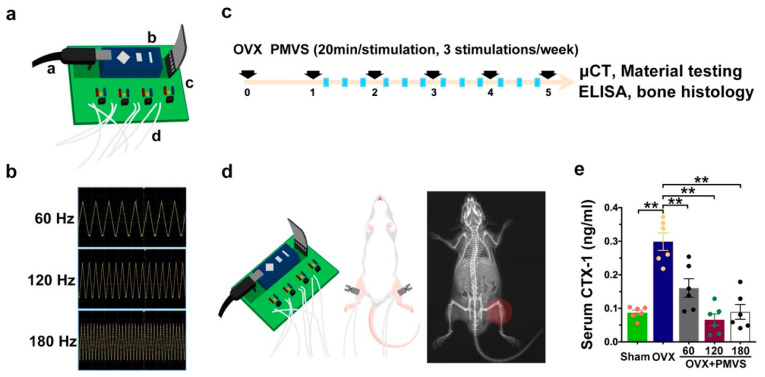
Effects of PMVS intervention on serum bone resorption marker levels in ovariectomized (OVX) mice. Scheme diagrams for PMVS device (a, microchip pulsed generator; b, amplifier; c, pulse wave modulator; d, piezoelectric vibration transducer) (**a**). Spectra of 60 Hz, 120 Hz, and 180 Hz PMVS with triangle waveform detected using laser displacement sensors (**b**). Experimental scheme (**c**) showing PMVS intervention onto distal femurs (**d**) of OVX mice for 4 weeks. PMVS interventions attenuated OVX-induced serum CTX-1 levels (**e**). Difference of investigations (mean ± S.E.; *n* = 5) were analyzed using Dunn’s nonparametric comparison test, where asterisks ** resemble *p* < 0.01.

We established a PMVS device (Figure 1a), including a microchip pulse generator, an amplifier, a pulse wave modulator, and 6 ceramic piezoelectric vibration transducers (1-cm in diameter). The device produced 60 Hz, 120 Hz, and 180 Hz vibration (waveform, triangle; amplitude, 0.05 g) (Figure 1b), which was calibrated using laser displacement sensor. Next to the PMVS device, we examined whether PMVS intervention affected the development of osteoporosis. To this end, we utilized ovariectomy (OVX)-induced bone loss in mice as a model of menopausal osteoporosis. One week postoperatively, ovariectomized mice were given PMVS (20 min/stimulation/day, 3 stimulations/week) for 4 consecutive weeks (Figure 1c). Animals were anesthetized and put in a supine position, ultrasound gel was put onto the skin around animals’ knees, and transducers were placed onto the gel around 5-mm above distal femurs (Figure 1d). All mice gained body weight. Animals’ activity was unaffected upon PMVS intervention. Ovariectomy significantly increased serum collagen I degradation product C-telopeptide of type I collagen (CTX-1), a bone resorption marker, as compared to sham operated mice. Sixty Hz, 120 Hz, and 180 Hz PMVS interventions repressed serum CTX-1 levels in OVX mice (Figure 1e). 

### 2.2. PMVS Intervention Preserved Bone Mass, Trabecular and Cortical Bone Microstructure 

**Figure 2:**
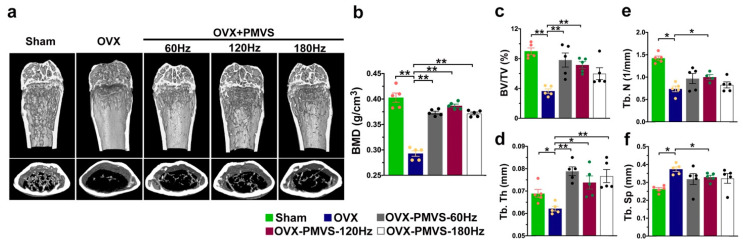
Quantitative µCT analysis of bone mass and trabecular and cortical microstructure. Sagittal and transverse views of µCT images showing mild loss in trabecular and cortical bone microarchitecture in OVX mice upon PMVS (**a**). Sixty Hz, 120 Hz, and 180 Hz PMVS attenuated OVX-induced loss in BMD (**b**), BV/TV (**c**), and Tb.Th (**d**). 120 Hz PMVS compromised OVX-induced Tb.N loss (**e**) and Tb.Sp (**f**). Difference of investigations (mean ± S.E.; *n* = 5) were analyzed using Dunn’s nonparametric comparison test, where asterisks * and ** resemble *p* < 0.05 and *p* < 0.01, respectively.

Sagittal and transverse views of µCT radiographs of distal femurs showed sparse trabecular bone networks and thin cortical bone microstructures in OVX mice, as compared to sham-operated mice. Skeletal tissue in 120 Hz and 180 Hz PMVS-treated OVX mice displayed mild loss in trabecular and cortical bone microstructure (Figure 2a). OVX reduced bone mineral density (BMD) (Figure 2b), trabecular volume (BV/TV; Figure 2c), trabecular thickness (Tb.Th; Figure 2d), and trabecular number (Tb.N; Figure 2e), whereas estrogen deficiency upregulated trabecular separation (Tb.Sp; Figure 2f) as compared to the sham operation group. PMVS interventions with 60 Hz, 120 Hz, and 180 Hz attenuated OVX-induced loss in BMD, BV/TV, and Tb.Th. One hundred and twenty Hz rather than 60 Hz or 180 Hz PVMS intervention compromised Tb.N and Tb.Sp of OVX skeletal tissue.

### 2.3. PMVS Intervention Attenuated OVX-Induced Biomechanics Inhibition

**Figure 3:**
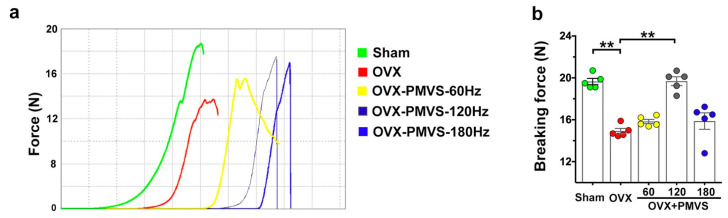
Analysis of biomechanical properties of bone tissue in sham operated and ovariectomized mice. Load-displacement profiles of femurs using three-point bending material test (**a**). One hundred and twenty Hz PMVS intervention attenuated OVX-induced loss in breaking force of femurs (**b**). Difference of investigations (mean ± S.E.; *n* = 5) were analyzed using Dunn’s nonparametric comparison test, where asterisks ** stand for *p* < 0.01.

Biomechanical strength together with bone mineral density and microarchitecture are prominent components of bone quality. We examined whether biomechanical properties of bone tissue in OVX mice were changed upon PMVS interventions. A material testing system was utilized to characterize load-displacement profiles and maximum breaking forces of bone specimens upon three-point bending (Figure 3a). Ovariectomy reduced breaking force of femurs, as compared to the sham operation group (Figure 3b). One hundred and twenty Hz rather than 60 Hz and 180 Hz PMVS intervention reversed OVX-mediated loss in biomechanical strength. Collective investigations of quantitative µCT analysis and material testing suggested that PMVS interventions were advantageous to slow the development of estrogen deficiency-mediated bone tissue deterioration. Of the magnitudes, 120 Hz PMVS intervention had the greatest protective effect on bone mineral density, microarchitecture, and biomechanical properties and was selected for subsequent experiments.

### 2.4. PMVS Intervention Improved Trabecular Morphology and Osteoclast Formation

**Figure 4:**
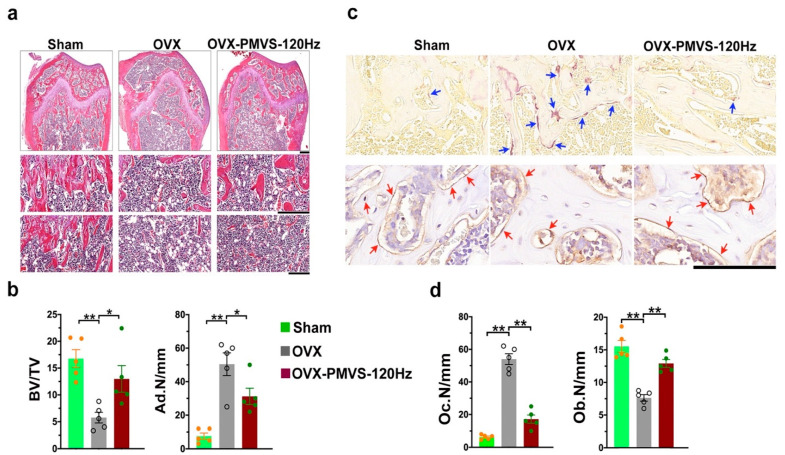
Histological analysis of trabecular bone morphology osteoclasts and osteoblasts in distal femoral bone specimens. Hematoxylin-eosin staining images revealing mild loss in trabecular bone loss (scale bar, 200 µm in upper panels; scale bar, 50 µm in middle panels) and moderate marrow adipocyte formation (scale bar, 50 µm in lower panels) in OVX bone tissue upon PMVS intervention (**a**). PMVS intervention reversed OVX-induced BV/TV loss and Ad.N upregulation (**b**). Tartrate-resistant acid phosphatase histochemical staining images (blue arrows) showing few osteoclasts and osteocalcin immunohistochemical staining (red arrows) images showing plenty of osteoblasts in OVX bone tissue upon PMVS intervention (**c**); scale bars, 10 µm. PMVS intervention attenuated OVX-induced osteoclast formation and osteoblast loss (**d**). Difference of investigations (mean ± S.E.; *n* = 4–5) were analyzed using Dunn’s nonparametric comparison test, where asterisks * and ** resemble *p* < 0.05 and *p* < 0.01, respectively.

Histologic features of osteoporotic skeletal tissue in OVX mice showed trabecular bone loss together with marrow adipocyte overdevelopment (Figure 4a) compared to the sham operation group, as evidenced in hematoxylin and eosin staining. PMVS-treated OVX bone specimens revealed moderate loss in trabecular bone morphology and mild fatty marrow (Figure 4a). Likewise, OVX impaired trabecular bone morphology integrity, as evidenced in significant decreases in trabecular bone volume/tissue volume (BV/TV) and increases in adipocyte number (Ad.N/mm) (Figure 4b). PMVS intervention compromised OVX-induced BV/TV loss and Ad.N upregulation in bone microenvironment. Furthermore, plenty of osteoclasts showing tartrate resistant acid phosphatase histochemical staining (Figure 4c) together with few osteoblasts, as evident from osteocalcin immunostaining, developed in the trabecular bone of OVX mice. Few osteoclasts and moderate loss in osteoblasts were present in skeletal tissue of OVX mice upon PMVS intervention (Figure 4c). Osteoclast number (Oc.N) was significantly increased in OVX bone specimens, whereas osteoblast number (Ob.N) was less than sham operated mice. PMVS intervention attenuated OVX-induced osteoclast overburden and osteoblast loss (Figure 4d), suggesting that PMVS intervention reversed OVX-mediated osteoclast overburden, repressing excessive bone turnover. 

### 2.5. PMVS Intervention Affected RANKL and OPG 

**Figure 5:**
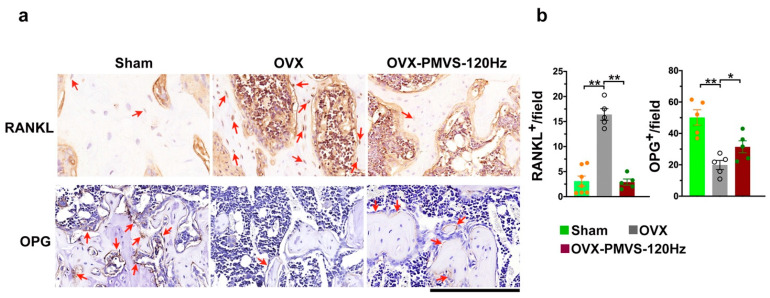
Immunohistochemical analysis of RANKL and OPG in bone tissue. Immunohistochemical staining images showing plenty of RANKL immunostained osteoblasts and few OPG immunostained osteoblasts in OVX bone tissue (red arrows) (**a**); scale bar, 10 µm. Osteoblasts in PMVS-treated bone specimens revealed weak RANKL and mild OPG immunoreactivity (**b**). Difference of investigations (mean ± S.E.; *n* = 4–5) were analyzed using Dunn’s nonparametric comparison test, where asterisks * and ** resemble *p* < 0.05 and *p* < 0.01, respectively.

Receptor activator of NF-kappaB ligand (RANKL) and osteoprotegrin (OPG) produced by bone-forming cells are important cytokines in bone remodeling reaction. The former cytokine in concert with macrophage-colony stimulating factor (M-CSF) is indispensable in osteoclastogenic differentiation of bone-marrow macrophage progenitor cells, and the latter inhibits osteoclast formation [3,5]. Given that PMVS intervention attenuated osteoclast overdevelopment in OVX-mediated osteoporotic skeleton, we wondered whether these cytokines in skeletal tissue microenvironment of OVX mice were changed upon PMVS intervention. In OVX bone specimens, osteoblasts lined along the trabecular bone and osteocytes in cortical lacuna showed strong RANKL immunostaining (Figure 5a) but displayed faint OPG immunoreactivity (Figure 5a) as compared to the sham operation group. Estrogen deficiency upregulated RANKL signaling but inhibited OPG immunoreaction (Figure 5b) in bone-making cells. In PMVS-treated bone specimens, osteoblasts and osteocytes showed slight RANKL immunostaining and moderate OPG immunoreaction (Figure 5b). OVX-induced RANKL and OPG signaling dysregulation was reversed in PMVS-treated skeletal tissue. Collective investigations of histomorphometry and immunohistochemistry suggested that PMVS intervention affected RANKL and OPG signaling in bone-making cells to repress estrogen deficiency-induced excessive osteoclastogenic activity and bone resorption reaction in the trabecular bone microenvironment.

### 2.6. PMVS Intervention Affected Wnt3a and Dkk1 Expression

**Figure 6:**
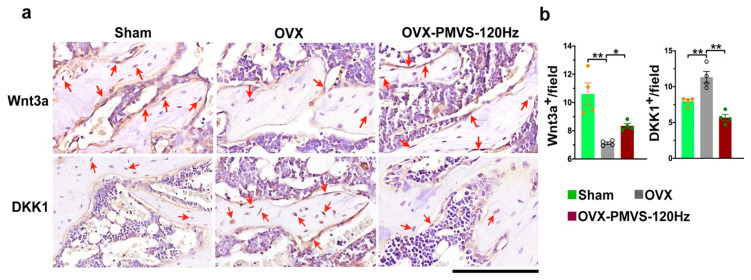
Immunohistochemical analysis of Wnt3a and Dkk1 in bone tissue. Immunohistochemical staining images showing few Wnt3a-immunostained osteoblasts (red arrows) and strong Dkk1 immunoreactivity in osteoblasts (red arrows) of OVX bone tissue. Osteoblasts in PMVS-treated bone revealed strong Wnt3a and weak Dkk1 immunoreactivity (**a**); scale bar, 10 µm. PMVS intervention attenuated OVX-induced Wnt3a loss and Dkk1 (**b**). Difference of investigations (mean ± S.E.; *n* = 4–5) were analyzed using Dunn’s nonparametric comparison test, where asterisks * and ** resemble *p* < 0.05 and *p* < 0.01, respectively.

Wnt3a and Wnt inhibitor Dkk1 play important roles in osteogenic activity and bone tissue homeostasis. The analysis of mild osteoblast loss in OVX bone tissue upon PMVS prompted us to investigate whether PMVS protection against OVX-induced bone loss correlated with Wnt3a or Dkk1 signaling. Immunohistochemistry uncovered that osteoblasts adjunct to trabecular bone in OVX mice showed weak Wnt3a immunostaining, whereas moderate Wnt immunoactivity was present in PMVS-treated bone specimens (Figure 6a). Osteoblasts in OVX bone displayed strong Dkk1 immunoactivity. This effect was repressed in PMVS-treated bone tissue intervention (Figure 6a). PMVS intervention significantly attenuated OVX-induced Wnt3a loss and Dkk1 overproduction (Figure 6b). 

### 2.7. PMVS Intervention Preserved Osteogenesis of Bone-Marrow Mesenchymal Cells

**Figure 7:**
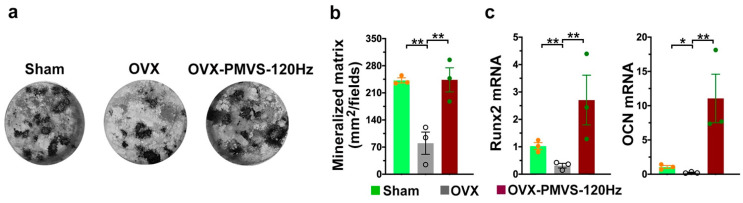
Analysis of osteogenic differentiation of primary bone marrow stromal cells. Von Kossa staining images showing mineralized matrix underproduction in the OVX group. This effect was improved upon PMVS intervention (**a**,**b**). PMVS intervention reversed mineralized matrix accumulation and osteogenic marker expression (**c**). Primary bone-marrow stromal cells were incubated in osteogenic media. Difference of investigations (mean ± S.E.; *n* = 3) were analyzed using Dunn’s nonparametric comparison test, where asterisks * and ** resemble *p* < 0.05 and *p* < 0.01, respectively.

We harvested primary bone-marrow stromal cells and investigated whether osteogenic activity in OVX bone microenvironment was altered upon PMVS intervention. Von Kossa staining revealed few mineralized nodules in OVX bone-marrow stromal cells as compared to the sham-operated mice. This effect was improved in OVX bone tissue upon PMVS intervention (Figure 7a,b). Likewise, OVX repressed osteogenic markers, including transcription factor Runx2 and extracellular matrix osteocalcin expression (Figure 7c). PMVS intervention improved OVX-induced loss in osteogenic marker expression of bone-marrow stromal cells. 

### 2.8. PMVS Intervention Promoted Osteogenic Gene Expression of MC3T3-E1 Osteoblasts

**Figure 8:**
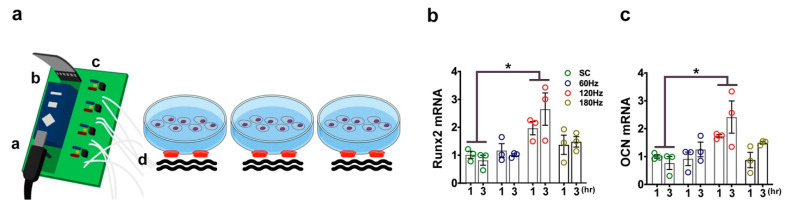
Piezoelectric microvibration stimulation (PMVS) intervention for MC3T3-E1 osteoblasts. Scheme drawing for PMVS intervention to MCT3-E1 osteoblasts incubated in cell culture dishes (a, microchip pulsed generator; b, amplifier; c, pulse wave modulator; d, piezoelectric vibration transducer) (**a**). PMVS increased Runx2 (**b**) and osteocalcin (**c**) expression. Difference of investigations (mean ± S.E.; *n* = 3) were analyzed using Dunn’s nonparametric comparison test, where asterisks * resemble *p* < 0.05.

We examined whether PMVS changed bone-forming cell activity. Murine MC3T3-E1 osteoblasts were incubated in cell culture dishes. Ceramic piezoelectric transducer was attached to the bottom of the cell culture dish using adhesive tape. Ultrasound gel was put in between the dish and the transducer (Figure 8a). Cell cultures were treated with 60 Hz, 120 Hz, or 180 Hz PMVS for 1 h or 3 h. Osteogenic marker expression, including transcription factor Runx2 (Figure 8b) and extracellular matrix osteocalcin (Figure 8c), were increased upon 120 Hz PMVS for 1 h and 3 h as compared to the control group, as evidenced in RT-PCR analysis. Osteogenic gene expression was unaffected upon 60 Hz or 180 Hz PMVS. These investigations suggested that PMVS promoted osteoblastic activity and 120 Hz PMVS had the greatest promotion for osteogenic gene expression and was selected for subsequent experiments. 

### 2.9. PMVS Intervention Promoted Mechanosensitive Signaling and Wnt Signaling in Osteoblasts

**Figure 9:**
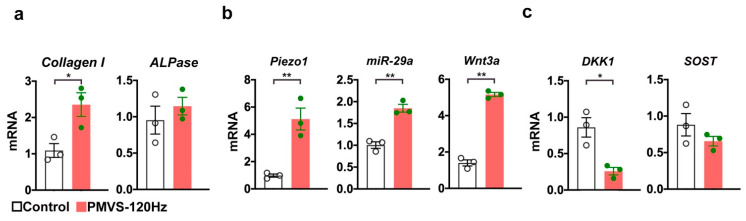
Effects of PMVS on bone marker, Piezo1, miR-29a, Wnt3a, Dkk1, and SOST expression in osteoblasts. One hundred and twenty Hz PMVS for 1 h promoted collagen I (**a**), Piezo1, miR-29a, and Wnt3a expression (**b**), whereas Dkk1 rather than SOST expression was repressed (**c**). Difference of investigations (mean ± S.E.; *n* = 3) were analyzed using the Wilcoxon test, where asterisks * and ** stand for *p* < 0.05 and *p* < 0.01, respectively.

Furthermore, bone matrix collagen I rather than bone alkaline phosphatase expression (Figure 9a) was also promoted in osteoblasts upon 120 Hz PMVS for 1 h. Expanding evidence has revealed that mechanosensitive molecule Piezo1 mediates bone-forming cell function and bone homeostasis upon biophysical stimulation [16,23]. MicroRNA-29a is indispensable in osteoblastic activity and protects skeletal tissue from estrogen deficiency-induced bone loss [24]. We uncovered that 120 Hz PMVS upregulated Piezo1 and miR-29a expression (Figure 9b). Consistent with the immunohistochemical analysis of bone specimens, PMVS promoted Wnt3a in osteoblasts (Figure 9b), whereas Wnt inhibitor Dkk1 rather than SOST expression was reduced (Figure 9c). 

## 3. Discussion

Mechanotransduction [25] or mechanical sensing [26] pathways are indispensable in maintaining survival, differentiation capacity, and mineralized matrix accumulation of bone-forming cells, as well as influence metabolism, remodeling, and regeneration of skeletal tissue [27]. Accumulating studies have revealed that biophysical intervention is advantageous to repress osteoporotic disorders or cancer-induced osteolytic diseases [28]. Preservation of bone anabolism for slowing down osteoporosis development using biophysical stimulation warrants investigation. We uncovered mild osteoporosis signs in oophorectomized mice upon piezoelectric microvibration stimulation (PMVS) intervention for 4 weeks. This intervention preserved osteogenic activity, which shielded skeletal microenvironment from estrogen deficiency-induced excessive bone turnover. Collective investigations highlight the perspective of a new biophysical remedy for delaying the development of osteoporotic disorders.

We established a PMVS intervention strategy and verified whether this biophysical stimulation promoted osteoblastic activity or had therapeutic potential for ovariectomized mice as an in vivo model of menopausal osteoporosis. The in vitro model revealed that increased osteogenic marker expression was present in MC3T3-E1 osteoblasts upon PMVS. While an accumulating number of reports of in vitro models have shown that vibration is advantageous to the mineralization capacity of biomaterials, little is understood about the development of estrogen deficiency-mediated osteoporosis upon PMVS intervention. For example, mechanical vibration (0.3 g, 30 Hz) for 8 weeks enhances porous titanium implant integration with bone tissue in diabetic rabbits [29]. Periodic vibration (0.3 g, 40 Hz) upregulates the osteoinduction capacity of biphasic calcium phosphate (BCP) scaffolds [30]. Low pulse vibration (0.25 N, 30 Hz) for 48 h reduces RANKL production of osteoblasts and increases osteoclastic marker cathepsin K levels in bone marrow cells [22]. In this study, quantitative µCT radiography and material testing analysis showed that local PMVS intervention (120 Hz, 0.05 g, 20 min/day) for 4 weeks had protective effect on bone mass, microarchitecture, and mechanical strength of ovariectomized mice, as well as downregulated osteoclast formation in osteoporotic bone tissue. Bone markers and RANKL expression in osteoblasts were also promoted upon PMVS for 1 h. We speculated that the biological response of bone-making cells to vibration appeared to depend on the stimulation magnitude, frequency, and time. To our best knowledge, this study is the first that sheds light onto a new electromechanical stimulation using very low vibration magnitude for delaying bone mass loss in 4 weeks and promoting osteoblastic activity in 1 h. 

PMVS intervention reversed osteoblast loss and osteoclast overburden to preserve trabecular morphology, suggesting that estrogen deficiency-induced excessive bone remodeling, a prominent histopathological feature of the osteoporotic skeleton [31], was attenuated in bone microenvironment of OVX mice. Accumulating studies of in vitro and in vivo models have uncovered that oscillatory mechanical stimulation changes osteoblastic activity or osteogenic differentiation of mesenchymal stem cells. Whole-body mechanical vibration intervention improves trabecular bone volume, marrow fat fraction, and trabecular BMD rather than stiffness of osteoporotic skeleton in menopausal women [18]. Oscillatory fluid flow promotes osteogenic lineage specification of mesenchymal stem cells [32]. The analysis of increased mineralized nodule formation and osteogenic gene expression of primary bone-marrow stromal cells in OVX mice upon PMVS consolidated the evidence that this intervention has an anabolic effect on bone tissue. 

However, the impact of biophysical vibration intervention on osteoclast function remains uncertain. A study of Sakamoto et al. has revealed that vibration induces growth rather than osteoclastogenic differentiation of RAW264.7 osteoclast precursor cells, whereas osteoclast formation is upregulated in osteoclast progenitor cells co-incubated with vibration-treated osteocytes in murine alveolar bone [33]. Low-intensity vibration downregulates osteoclastogenic differentiation of bone-marrow monocytes in spinal injury-induced osteoporotic bone [34]. In this study, PMVS intervention attenuated OVX-mediated osteoclast overdevelopment and Tb.Sp. We speculated that the effect of vibration on osteoclast formation may depend on bone tissue type or vibration intensity. Investigations of weak RANKL immunostaining in osteoblasts of OVX bone tissue and decreased serum CTX-1 levels upon PMVS explained an anti-resorption reaction in skeletal tissue upon the intervention. 

We revealed that Piezo1, miR-29a, and Wnt3a signaling were molecular mechanisms underlying PVMS intervention promotion of osteoblastic activity, which delayed OVX-induced bone loss. Piezo1, a mechanosensitive molecule, is required to maintain bone tissue integrity and convert extracellular biophysical stimuli into intracellular signaling [35]. Osteoblast-specific Piezo1 knockout mice show severe osteoporosis and have less response to biophysical stimulation of bone formation [36]. miR-29a plays an important role in maintaining bone formation activity of osteogenic cells [37]. Osteoblast-specific miR-29a transgenic mice develop less osteoporosis than ovariectomized wild-type mice [24]. Wnt and Wnt inhibitors, including GSK-3β, Dkk-1, and SOST, are important pathways that transduce biophysical stimulation into intracellular signaling [38]. The pulsed electromagnetic field increases Wnt1 and Wnt10b signaling, which promotes bone regeneration in rats with spinal injuries [39]. Mechanical stimulation upregulates Twist, Dkk1, and SOST, increasing cortical bone mass [40]. Whole-body vibration activates Wnt and downstream effector β-catenin signaling, stimulating bone formation in young rats [41]. Furthermore, miR-29a is found to target Dkk-1 expression, promoting osteogenic activity [42]. The analysis of miR-29a expression underpinned the data of decreased Dkk1 expression in bone-forming cells upon PMVS. This study revealed that PMVS intervention appeared to activate Piezo1 signaling, which increased Wnt and miR-29a signaling to promote osteoblastic activity. The possibility cannot be excluded that other mechanoresponsive molecules may involve PMVS improvement of bone integrity. The limitation of this study is that the PMVS intervention prevented bone loss in mice 1 week upon ovariectomy; however, the ubiquitous or long-term effect of this intervention on the development of estrogen deficiency or age-induced osteoporosis warrants investigation. 

Taken together, PMVS intervention promotes Piezo1, miR-29a, and Wnt3a signaling, which upregulate osteogenic gene expression and mineralization matrix formation in bone-forming cells. These effects are advantageous to regulate RANKL and OPG signaling to repress estrogen deficiency-induced excessive bone resorption and delay bone mass and quality loss. This study sheds light onto a new biophysical mechanical intervention for skeletal tissue and highlights the remedial potential of PMVS intervention for preventing against osteoporotic diseases. 

## 4. Materials and Methods

### 4.1. Experimental Osteoporosis Models 

Laboratory animals use and research protocols were reviewed and approved (Affidavits 2018091703, 2017092802) by Institutional Animal Care and Use Committee, Kaohsiung Chang Gung Memorial Hospital. Female C57L/B6 mice (12 weeks old) were randomly divided into 5 groups. Upon anesthesia, mice were oophorectomized or sham operated without removing bilateral ovaries using aseptic surgical procedures as previously described [24]. Seven days postoperatively, ovariectomized mice were given PMVS interventions. 

### 4.2. PMVS Intervention 

A PMVS device, including a microchip pulse generator (Microchip Technology Inc. Chandler, AZ, USA), a micro-amplifier (Texas Instruments, Dallas, TX, USA), a pulse wave modulator (Texas Instruments, Dallas, TX, USA), and 6 ceramic piezoelectric vibration transducers (1-cm in diameter; Steiner & Martins, Inc., Davenport, FL, USA) were prepared. The device produced 60 Hz, 120 Hz, and 180 Hz vibrations (waveform, triangle; amplitude, 0.05 g). Vibration magnitude and frequency were calibrated using a spectrum analyzer (Agilent Technologies Inc., Santa Clara, CA, USA) and laser displacement sensors (Micro-Epsilon Messtechnik, Ortenburg, Germany). Ovariectomized mice were anesthetized using inhale anesthetic agents and placed in a supine position. Upon shaving around the knees, ultrasound gel (AQUASONIC CLEAR^®^; Parker Laboratories, Inc., Fairfield, NJ, USA) was put onto the skin around the knees. A ceramic piezoelectric vibration transducer was put 5-mm above the knee of femurs. Each femur was given PMVS (20 min/stimulation, 3 stimulations/week) for 4 consecutive weeks. All animals were euthanatized at the end of study. Venous blood was harvested, and bone specimens were dissected. 

### 4.3. In vitro PMVS Treatment

Murine MC3T3-E1 osteoblasts (3 × 10^5^ cell/dish) were incubated in cell culture dishes (3.5-cm in diameter; Thermo Scientific™ Nunc™ EasYDish™; Thermo Fisher Scientific, Inc., Waltham, MA, USA) with DMEM and 10% fetal bovine serum till 80% confluence. A PMVS Ceramic piezoelectric transducer was attached to the bottom of each cell culture dish using adhesive tape. Sixty Hz, 120 Hz, or 180 Hz PMVS was applied to each dish for 1 h or 3 h. Cell cultures incubated in dishes without PMVS were designated in controls. All protocols were performed in a 5% O_2_, 37 °C humidified incubator. 

### 4.4. Quantification of Serum CTX-1

Serum bone resorption marker C-telopeptide of type I collagen (CTX-1) was investigated using Mouse CTX-1 ELISA Kits (MBS9141384, MyBiosources, San Diego, CA, USA), according to the maker’s manuals. In brief, 100 µL serum was pipetted in a precoated well plate, mixed with 50 µL Sample Solution, and incubated at 37 C for 1 h. Upon washing with Wash Solution, 50 µL Solution A and 50 µL Solution B were added to each well. Optical density of the mixture was read using spectrophotometry with 450 nm wavelength. Serum CTX-1 levels were calculated using a standard curve of 0–10 ng/mL CTX-1 authenticate and calculated using Prism software (GraphPad, San Diego, CA, USA).

### 4.5. Quantitative µCT Analysis

Microcomputed tomography (μCT) radiography of bone specimens and reconstruction of 400 radiographs (9-µm pixel) into sagittal and transverse views of images were performed using 1176 Skyscan scanner (Bruker, Billerica, MA, USA) and SKYSCAN^®^ CT-Analysis software, according to producer’s instructions. The bone mineral density of the region of interest (ROI) was measured upon calibration using hydroxyapatite phosphate (HA) phantom (750 mgHA/cc, Computerized Imaging Reference Systems, Inc., Norfork, VA, USA). Morphometry of ROI, including BMD (mg/cm^3^), BV/TV (%), Tb.Th (mm), Th.N (1/mm), and Th.Sp (1/mm) was calculated using the software, as previously described [24,43]. 

### 4.6. Biomechanical Analysis

A 3-point bending (6-mm jag span) protocol was utilized to characterize biomechanical properties, including load-displacement profile and breaking forces, of femurs using SHIMADZU EZ-SX Material Test System (Shimadzu Corporation, Kyoto, Japan), as previously described [24]. The displaced speed of stainless-steel load (50 N) was set at 2 cm/min using TRAPEZIVMX software.

### 4.7. Histology

Five-micromillimeter sections of paraffin-embedded specimens were prepared and hematoxyline-eosin stained (ab245880, Abcam, Cambridge, UK), as previously described [40]. Trabecular morphology was evaluated at ×125 magnification using Axio Imager M2 light/fluorescence microscope (Carl Zeiss, Oberkochen, Germany). Trabecular bone volume (BV) and tissue volume (TV) of each field were measured using the AxioCam HRc image analysis system (Carl Zeiss, Oberkochen, Germany). Osteoblasts and osteoclasts in sections were investigated using osteocalcin immunohistochemical staining and tartrate-resistant acid phosphatase staining Kits (B-Bridge International Inc., Mountain View, CA, USA), respectively. 3 fields of each section and 3 sections were randomly selected for evaluation. Trabecular bone volume (BV/TV, %), osteoblast number (Ob.N), and osteoclast number (Oc.N) were calculated, according to the histomorphometry guideline of the American Society for Bone and Mineral Research [44]. 

### 4.8. Immunohistochemisty

Immunostaining of sections was investigated using RANKL (ab216484; Abcam, Cambridge, UK), OPG (ab203061; Abcam Cambridge, UK), Wnt3a (1H12L14, Thermo Fisher Scientific, Inc., Waltham, MA, USA), and Dkk1 (BS-2162R; Thermo Fisher Scientific, Inc., Waltham, MA, USA) antibodies and All-in-One Kit for Immunohistochemical Staining for Tissues (Thermo Fisher Scientific, Inc., Waltham, MA, USA). Immunostained osteoblasts were counted using light microscope and image analyzer (Carl Zeiss, Oberkochen, Germany). 3 fields of each section and 3 sections were randomly selected for evaluation.

### 4.9. Ex Vivo Osteogenesis of Primary Bone-Marrow Stroma Cells

Primary bone-marrow cells were isolated from femurs and mixed with RBC lysis buffer (11814389001; Sigma-Aldrich, Merck KGA, Darmstadt, Germany) to collect mononuclear cells. Upon incubating mononuclear cells in medium (DMEM with 10% fetal bovine serum; Gibco) overnight, adherent cells were harvested and incubated (10^5^ cells/well, 24-well plates) in osteogenic medium (StemPro™ Osteogenesis Differentiation Kit; Thermo Fisher Scientific, Inc., Waltham, MA, USA) for 18 days. Mineralized nodule formation was investigated using von Kossa Stain Kits (ab150687; Abcam Cambridge, UK), according to maker’s instruction. Von Kossa-stained areas in each field (mm^2^/filed) was measured using light microscope and image analyzer. 

### 4.10. RT-PCR

mRNA expression in MC3T3-E1 osteoblasts or in primary bone-marrow stromal cells were quantified using RT-PCR protocols with customized primers Runx2 (forward: 5′-CCAGCAGCACTCCATATCTC-3′; reverse, 5′-CAGCGTCAACACCATCATTC-3′), osteocalcin (forward, 5′-CAAGCAGGGAGGCAATAAGG-3′; reverse, 5′-CGTCACAAGCA GGGTTAAGC-3′), collagen I (forward, 5′-TAACTACGCGATGCTAA-3′; reverse, 5′-TTT AACGGTACTAGGA-3′); bone alkaline phosphatase (forward, 5′-AATCTAGATCAG GCTAGT-3′; reverse, 5′-GTACTGAATCGATGAATCG-3′; reverse 3′-ATTGCTATGCGTACCGGT-3′); Piezo1 (forward, 5′-TACTTAAGCTAGCAATC-3′ reverse, 5′-AAGTCA GGTCAGCTATC-3′) Wnt3a (forward, 5′-GATCAAGGTACGGTACG-3′; reverse, 5′-TTA AGTAACCGTACC-3′); Dkk1 (forward, 5′-CCGAATTAGGCCGATGTTCA-3′; reverse, 5′-TTACGAA GTTACGATCA-3′) SOST (forward, 5′-CCTACGTACAAGTCA-3′; reverse, 5′-ATGGAA CTGCTAGCTCC-3′), β-actin (forward: 5′-GACGGCCAGGTCATCACTAT-3′; reverse, 5′-CTTCTGCATCCTGTCAGCAA-3′), miR-29a (5′-UCACAGAACCGGUCUCUUU-3′), and U6 (5′-GTGCTCGCTTCGGCAGCACATATACTAAAATTGGAACGAT ACAGAGAAGATTACATGGCCCCCGCAGGATGACACGCAAATTCGTGAAGCGTT CCATATTTT-3′), SuperScript™ One-Step RT-PCR System with Platinum™ Taq DNA Polymerase (Thermo Fisher Scientific, Inc., Waltham, MA, USA) and ABI 7900 Detection System (Applied Biosystems, Foster City, CA, USA). Relative fold changes of mRNA expression were calculated using 2^−ΔΔCt^ [24,44].

### 4.11. Statistical Analysis

Investigations (mean ± standard error) were calculated using Prism software (GraphPad, San Diego, CA, USA). Differences of sham, OVX, and OVX-PMVS groups were analyzed using Dunn’s nonparametric comparison test. Differences of control and PMVS in MC3T3-E1 osteoblasts were analyzed using the Wilcoxon test using SPSS Statistics Software. *p* < 0.05 stands for significant difference between investigations. 

## Data Availability

The data that support the findings of this study are available on request from the corresponding author.
